# Walking speed related joint kinetic alterations in trans-tibial amputees: impact of hydraulic 'ankle’ damping

**DOI:** 10.1186/1743-0003-10-107

**Published:** 2013-10-17

**Authors:** Alan R De Asha, Ramesh Munjal, Jai Kulkarni, John G Buckley

**Affiliations:** 1 Division of Medical Engineering, School of Engineering, University of Bradford, Bradford BD7 1DP, UK; 2 Mobility & Specialised Rehabilitation Centre, Northern General Hospital, Sheffield S5 7AT, UK; 3 Disablement Services Centre, University Hospital of South Manchester, Manchester M20 1LB, UK

**Keywords:** Amputee, Ankle damping, Gait, Moments and powers, Prosthetic, Walking speed

## Abstract

**Background:**

Passive prosthetic devices are set up to provide optimal function at customary walking speed and thus may function less effectively at other speeds. This partly explains why joint kinetic adaptations become more apparent in lower-limb amputees when walking at speeds other than customary. The present study determined whether a trans-tibial prosthesis incorporating a dynamic-response foot that was attached to the shank via an articulating hydraulic device (*hy*A-F) lessened speed-related adaptations in joint kinetics compared to when the foot was attached via a rigid, non-articulating attachment (*rig*F).

**Methods:**

Eight active unilateral trans-tibial amputees completed walking trials at their customary walking speed, and at speeds they deemed to be slow-comfortable and fast-comfortable whilst using each type of foot attachment. Moments and powers at the distal end of the prosthetic shank and at the intact joints of both limbs were compared between attachment conditions.

**Results:**

There was no change in the amount of intact-limb ankle work across speed or attachment conditions. As speed level increased there was an increase on both limbs in the amount of hip and knee joint work done, and increases on the prosthetic side were greater when using the *hy*A-F. However, because all walking speed levels were higher when using the *hy*A-F, the intact-limb ankle and combined joints work per meter travelled were significantly lower; particularly so at the customary speed level. This was the case despite the *hy*A-F dissipating more energy during stance. In addition, the amount of eccentric work done per meter travelled became increased at the residual knee when using the *hy*A-F, with increases again greatest at customary speed.

**Conclusions:**

Findings indicate that a trans-tibial prosthesis incorporating a dynamic-response foot reduced speed-related changes in compensatory intact-limb joint kinetics when the foot was attached via an articulating hydraulic device compared to rigid attachment. As differences between attachment conditions were greatest at customary speed, findings indicate a hydraulic ankle-foot device is most effectual at the speed it is set-up for.

## Background

The determination of muscle moments and associated powers at the joints of the lower limbs provides key insights in to what, mechanically, is driving locomotion. When walking at their customary speed over level ground, able-bodied individuals typically display a period of low-magnitude power absorption at the ankle joint, for the first three quarters of stance, at which point a period of larger magnitude power generation occurs [1-3]. At the hip, moderate magnitude power is generated during early and late stance with a short period of power absorption at mid-stance [4,5]. In contrast, the knee tends to predominantly absorb power throughout stance with very little power generation occurring [3,4,6]. When walking at their customary speed unilateral trans-tibial amputees (UTAs) compensate for the absent foot and ankle by increasing early and late stance power generation at both hips and increasing late stance power generation at the intact ankle [7-11]. Despite such compensations, UTAs tend to have a slower freely chosen walking speed than able-bodied persons [12]. In addition, the moments and powers (peaks and integrals) at the residual knee are reduced compared to the intact side [7,13,14], most likely due to a desire to minimise loads on the residuum.

To increase walking speed, able-bodied individuals predominantly increase stance-phase power generation at the hip (and moderately increase power generation at the ankle) [5,15,16]. They also display increases in both power absorption and generation at the knee [5,15]. Thus as walking speed increases, the proportional contribution of the ankle to gait propulsion reduces while the contributions of the hips and knees increase [5]. In UTAs walking speed is likewise increased by increasing stance-phase power generation at the hip on both the intact and prosthetic sides [5].

The purpose of a prosthetic ankle-foot device is to approximate the function provided by the absent physiological structures. Modern, passive prosthetic devices typically incorporate flexible keels that are able to absorb and return power during stance through elastic deformation and recoil. Such deformation occurs irrespective of whether the foot is fixed to the prosthetic shank rigidly (non-articulating) or via a device allowing articulation (e.g. MultiFlex 'ankle’ uses a rubber snubber at the point of attachment). Not surprisingly, use of a dynamic response (sometimes referred to as energy-storing and return) foot compared to semi-rigid foot (e.g. SACH) has been shown to increase the amount of late stance power returned at the prosthetic 'ankle’ as well as power absorbed at the residual knee [17].

Prosthetic ankle-foot devices are typically aligned and set-up to provide optimal function at the user’s customary walking speed. This implies their function will be sub-optimal at higher or lower speeds. Recently an ankle-foot device incorporating a hydraulically controlled (passive) articulating attachment has become clinically available (Echelon, Chas. A. Blatchford & Sons, Ltd., Basingstoke, UK). This device allows nine degrees of damped, and thus time-dependent, articulation between the foot (dynamic-response) and shank. Use of this device by active, UTAs, has been shown to result in reduced in-socket pressures [18], a less disrupted centre-of-pressure (CoP) progression [19] and an increased freely chosen walking speed [19]. The device is set-up, for customary speed walking, by adjusting the hydraulic resistance thus altering the rate at which articulation occurs. Because of the controlled articulation provided by the hydraulic unit, it can passively realign (tilt) in the sagittal plane when walking on slopes. This ability to change alignment may also accommodate changes in walking speed because of the requirement of the foot / ankle to go through a greater range of motion when walking at faster speeds and thus use of the device may offer speed-related advantages over more traditional attachment types. The purpose of the present study was to investigate whether a trans-tibial prosthesis incorporating a dynamic-response foot that was attached to the shank via an articulating hydraulic device (*hy*A-F) reduced speed-related adaptations in joint kinetics compared to when the foot was attached via a rigid, non-articulating attachment (*rig*F). Specifically, sagittal plane joint moments and powers were compared between attachment categories when UTA participants walked at their freely chosen slow, customary and fast speeds. It was hypothesized that due to having fewer and / or smaller disruptions in CoP progression under the foot during prosthetic limb stance when using a *hy*A-F [19] the speed-related increases in compensatory stance-phase power generation at the hip (both limbs) and ankle (intact limb) would be reduced. It was further hypothesized that due to the dampened ankle articulation offered by the *hy*A-F there would be increased loading / involvement of the residual knee across all speeds.

## Methods

### Participants

Eight male, physically active UTAs (mean ± SD age 44.8 ± 10.7 years, mass 83.3 ± 19.0 kg, height 1.77 ± 0.05 m) took part, each giving written informed consent prior to their involvement. All had undergone amputation at least two years prior to participation (mean 6.7 ± 5.5 years, range 2 to 19 years) and all had used their current prosthesis for at least six months. All participants habitually used an Esprit foot (Chas. A. Blatchford and Sons Ltd., Basingstoke, UK). This foot is identical in design to a *hy*A-F except that it has a non-articulating, 'rigid’ attachment (*rigF*).

The study was conducted in accordance with the tenets of the Declaration of Helsinki and approval was gained from the University of Bradford’s Committee for Ethics in Research.

### Protocol and prosthetic intervention

Participants completed overground walking trials along a flat and level 8 m walkway at three different speed levels: customary, comfortable 'slow’ and comfortable 'fast’. Participants were instructed to walk “at their normal walking speed”, “slowly” and “as fast as comfortably possible”. Trials were undertaken in two blocks, each made up of sets of trials at each of the three walking speed levels. One block was undertaken using the *rig*F and the other using a *hy*A-F. Attachment type order was counterbalanced across participants as was the order of 'fast’ or 'slow’ sets, following the customary speed set which was always performed first. A successful trial occurred when a 'clean’ contact by the prosthetic or intact foot was made with either of two floor mounted force platforms without any observable targeting or changes in stride pattern. Trials were repeated until 10 'clean’ contacts of each foot were made at each speed in each attachment condition. Due to the counter-balanced experimental design and because of the methodological limitations associated with speed-controlled studies and the difficulty in generalising findings from such studies to the natural environment [20] we decided not to control walking speed across attachment conditions. Prior to completing the block using the *hy*A-F each participant’s prosthesis was altered by exchanging the existing *rig*F device for a *hy*A-F. All alterations were made by the same experienced prosthetist. Everything about the prosthesis was kept as near to constant as possible when one attachment type was exchanged for the other. The socket, suspension, overall length of the prosthesis and alignment of the shank pylon were unchanged across attachment types. When swapping from a *rig*F to a *hy*A-F, or vice versa, the foot would naturally fall into the existing alignment and only shank length was adjusted by either shortening or lengthening the pylon.

The *hy*A-F has separate damping settings for plantar- and dorsi-flexion ranging from 1 [minimum] to 9 [maximum], which equate to damping coefficients of 1.28 to 3.48 Nm.s/deg respectively. Once the *hy*A-F was fitted, participants used the prosthesis both indoors and outdoors for a minimum of 45 minutes prior to data collection for accommodation. They negotiated ramps, slopes and stairs and walked over a variety of surfaces including pavements, grass verges and carpeted floors. At the beginning of this period the settings which control the rates of articulation within the *hy*A-F were adjusted by the prosthetist until deemed to provide “optimal function” at self-selected, customary walking speed. The adjustment consisted of systematically altering the levels of damping of both plantar- and dorsi-flexion while each participant walked using the device. The final settings were decided upon using a mixture of participant feed-back regarding perceived comfort and function and the prosthetist’s experience. Participants completing trials using the *rig*F first (block 1) completed these on arrival at the laboratory. For those completing trials using the *rig*F second (block 2) the foot was refitted to their prosthesis following completion of block 1 and the original condition of the prosthesis returned. Participants were again given an accommodation period, similar to that described above, in order to reacquaint themselves with their habitual prosthesis prior to data collection.

### Data acquisition

Kinematic and kinetic data were recorded at 100 Hz and 400 Hz respectively using an eight camera motion capture system (Vicon MX, Oxford, UK) and two force platforms (AMTI, MA, USA) using methods described previously [19]. A 6DoF model was used to determine segmental kinematics [21]. Such an approach avoids having to position markers over joint centres where soft-tissue movement might otherwise cause movement artefact [22,23]. It also facilitates the method used to locate functional joint centres for the lower-limbs (details below). Determination of a functional joint centre for the residual knee was of particular importance because this joint was enclosed within the socket, which made palpation and accurate identification of the femoral condyles extremely difficult, if not impossible. The segments tracked were the head, thorax / abdomen, pelvis and left and right thighs, shanks and feet. Motion of the arms was not monitored. Labelling and gap filling of marker trajectories were undertaken within Workstation software (Vicon, Oxford, UK). The C3D files were then exported to Visual 3D motion analysis software (C-Motion, Germantown, MD, USA), where all further processing took place.

Firstly, data (kinematic, kinetic) were filtered using a forth order, zero-lag Butterworth filter with a 6 Hz cut-off. The period of stance was then determined: initial contact and toe-off were defined as the instants the vertical component of the ground reaction force (GRF) first went above or below 20 N respectively. Stance phase lower-limb joint kinetics were then determined as follows.

### Biomechanical modelling

The dynamic-response foot, which is integral to both the *hy*A-F and *rig*F, has flexible heel and forefoot keels which, when loaded, deform simulating plantar- and dorsi-flexion about non-defined axes. Thus, as with all such feet, the assumptions of a rigid segment and pin joint articulation [24] are violated. Consequently, the assessment and interpretation of 'ankle’ kinetics can be problematic and sometimes misleading [25-27]. Therefore we chose not to use a standard inverse dynamics approach [24] to calculate 'ankle’ kinetics for the prosthetic limb. Instead we used a validated [28] and previously used [29-32] methodology to estimate the energy absorbed and returned by the prosthetic foot by determining the sagittal plane power flow at the distal end of the prosthetic shank (*pros end). Pros end* was defined on the (longitudinal) segment mid-line at the same height as the contralateral intact ankle. This definition was used in both foot attachment conditions to enable valid (unbiased) comparisons between them. Regardless of the type of attachment and / or foot, *pros end* is the physical application point of the forces and moments transferred to and from the shank. As such this modelling approach, as the authors highlighted [28], can be used for either articulating or non-articulating ankle-foot devices.

**Figure 1:**
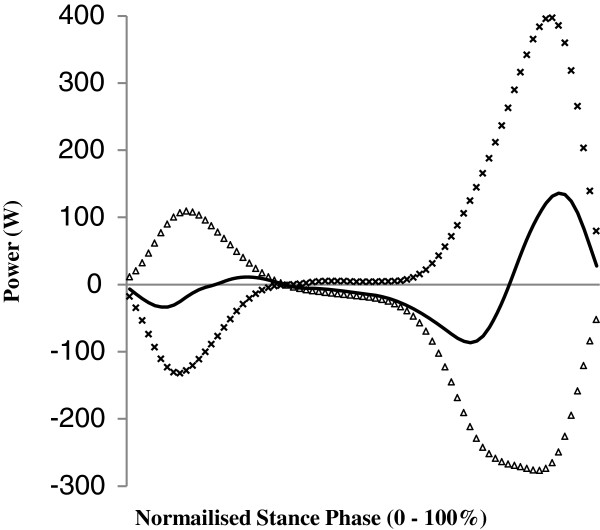
**Exemplar trial showing stance phase net power (P**_**dist**_**, solid line) along with the rotational prosthetic power(P**_**rot**_**, triangles) and translational joint power (P**_**trans**_**, crosses) at the distal end of the prosthetic shank (*pros end*).** Negative values indicate power leaving the shank (flow to the foot) and positive values indicate power entering the shank (flow from the foot).

Therefore the energy entering or leaving the prosthetic foot was assessed by summing the sagittal plane translational and rotational power flows at the *pros end* (Figure 1), as per the method described by Prince et *al*. [28].

Translational joint power (P_trans_) was defined as:

(1) $$P_{\mathrm{trans}}=\left(F_{z}.V_{z}\right)+\left(F_{y}.V_{y}\right)$$

Where; F_y_ and F_z_ are the antero-posterior and vertical components of the reaction force (N) acting at *pros end* and V_y_ and V_z_ are the antero-posterior and vertical velocities of *pros end* (m.s^-1^).

Rotational prosthetic power (P_rot_) was defined as:

(2) $$P_{\mathrm{rot}}=M_{x}.\omega_{s}$$

Where; M_x_ is the sagittal moment acting at *pros end* (Nm) and ω_s_ is the angular velocity of the shank segment (rad.s^-1^).

Net power (P_dist_) at the *pros end* was calculated as:

(3) $$P_{\mathrm{dist}}=P_{\mathrm{trans}}+P_{\mathrm{rot}}$$

Thus the time integral of negative power over the stance phase yielded the energy leaving the shank and flowing to the prosthetic foot (P_dist_^(neg)^) while the time integral of the positive power yielded the energy entering the shank and flowing from (returned by) the prosthetic foot (P_dist_^(pos)^) (Figure 1).

All physiological joint centres (both hips, both knees, and the intact ankle) were located using a functional joint centre approach [33]. For the residual knee, this approach correctly located the sagittal joint axis but identified the joint centre located towards the lateral femoral condyle. We reasoned that this was due to movement between the prosthetic socket and residuum when the limb was flexed and extended during the non-weight bearing movement trial undertaken to determine the functional knee joint centre. Thus the joint centre was moved along the sagittal axis to a point equidistant from the medial and lateral knee markers. Joint centre virtual landmarks were used to define the endpoints of the associated segments. Sagittal plane joint kinetics (muscle moments and associated powers) at all physiological joints were calculated using standard inverse dynamics [24]. At the residual knee, joint kinetics were determined by assuming the foot and shank to be a single rigid segment with the distal forces acting on the segment being the GRF [34].

### Data processing and analysis

All moment and power data were normalised to body weight. Based on previous findings [19] we expected that the slow, customary and fast walking speed levels would all be higher when using the *hy*A-F than when using the *rig*F. As such, we also normalised all moment and power measures to walking speed [16,35]. Thus, in addition to absolute differences, the results presented indicate differences per metre travelled. Walking speed was determined as the average forwards (antereo-posterior) velocity of the whole-body centre of mass (COM) through the capture volume. The location of the whole-body COM was determined as the weighted average of the nine tracked segments [36].

The effects of attachment category and speed level on the absolute and speed-normalised moments and powers at each joint where determined by comparing joint moment peaks, and joint power peaks and power integrals (work) across conditions. Negative and positive power integrals were assessed separately to provide an insight, respectively, of the eccentric and concentric work (intact joints) or energy absorbed and returned by the prosthetic foot. The scalar magnitudes of these integrals were also then summed to yield the total work done at each physiological joint for both the intact and residual limbs. The difference between negative and positive power integrals at the *pros end* was calculated to yield the net energy dissipated by the ankle-foot device.

All parameters of interest were calculated for each individual trial and then averaged across trials to give a mean value for each participant, at each speed, in each attachment condition.

### Statistical analyses

Due to the different modelling approaches used across the intact and prosthetic limbs, no inter-limb comparisons were made. Instead we focussed on determining intra-limb changes as a result of a change in foot attachment condition. Comparisons between foot attachment conditions were undertaken using repeated measures ANOVA with attachment type (*hy*A-F, *rig*F) and speed level (slow, customary and fast) as repeated factors. Where main effects were significant post hoc analyses were conducted using Tukey HSD tests. Statistical analyses were made using Statistica (StatSoft, Inc., Tulsa, OK, USA). The alpha level was set at 0.05.

## Results

Walking speed was significantly affected by attachment category (*p* = 0.014) and by speed level (*p* < 0.001) but there was no interaction between terms (*p* = 0.053). The three speed levels were significantly different from each other (p ≤ 0.003), and all were higher when using the *hy*A-F but post-hoc analysis indicated speed differences between attachment types were only significant for the customary speed level (slow; *hy*A-F 0.95 ± 0.14 ms^-1^, *rig*F 0.93 ± 0.11 ms^-1^, *p* = 0.8, customary; *hy*A-F 1.18 ± 0.18 ms^-1^, *rig*F 1.09 ± 0.19 ms^-1^, *p* = 0.016, fast; *hy*A-F 1.38 ± 0.26 ms^-1^, *rig*F 1.36 ± 0.26 ms^-1^, *p* = 0.9).

### Joint kinetics

**Figure 2:**
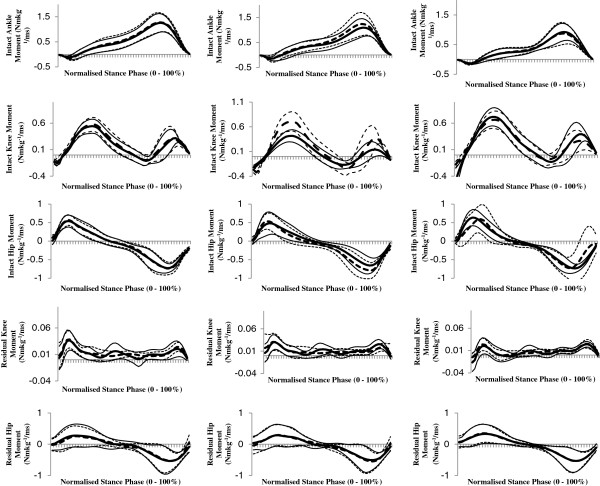
**Ensemble mean (± 1 SD) speed normalised stance phase moments at the hip, knee and ankle (intact limb only) joints when using rigid (*rig*** **F, dotted lines) and hydraulic (*hy*** **A-F, solid lines) ankle-foot devices at slow (left column), customary (centre column) and fast (right column) speed levels.**

**Figure 3:**
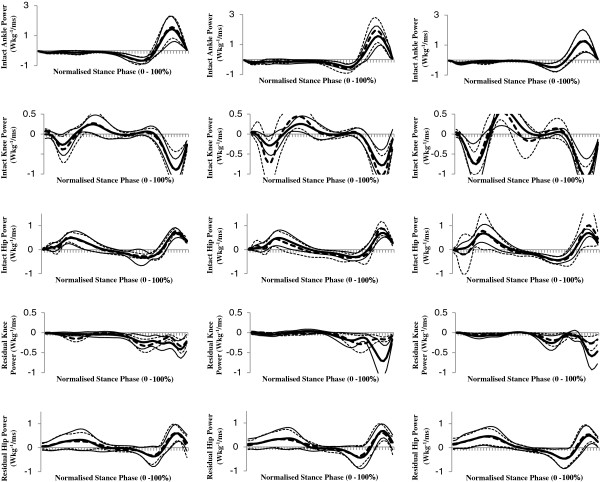
**Ensemble mean (± 1 SD) speed normalised stance phase muscle power generation (positive) and absorption (negative) at the hip, knee and ankle (intact limb only) joints when using rigid (*rig*** **F, dotted lines) and hydraulic (*hy*** **A-F, solid lines) ankle-foot devices at slow (left column), customary (centre column) and fast (right column) speed levels.**

**Figure 4:**
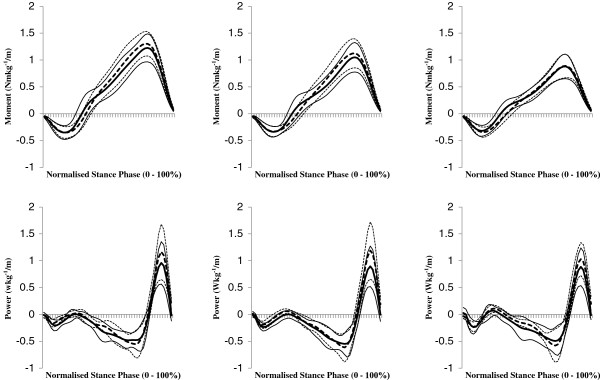
**Ensemble mean (± 1 SD) speed normalised stance phase external moment (plantarflexion – negative, dorsiflexion – positive) and power profiles at *pros end *when using rigid (*rig*F, dotted lines) and hydraulic (*hy*A-F, solid lines) ankle-foot devices at slow (left column), customary (centre column) and fast (right column) speed levels.** Negative power is power leaving the shank into the foot and positive power is power returning to the shank from the foot.

As we were not interested in speed-effects *per se,* the 'in text’ results only detail the effects of attachment category and / or attachment category by speed interactions. In addition, to avoid repetition, only speed-normalised results are detailed. Speed main effects are indicated in the results tables (Tables 1, 2 and 3); which present data both as speed normalised and in absolute terms. Ensemble average, speed-normalised joint moments and powers for physiological joints of both limbs are shown in Figures 2 and 3, and for *pros end* in Figure 4.

**Table 1:** **Group mean (± SD) peak extension (positive) and flexion (negative) muscle moments and concentric (positive) and eccentric (negative) muscle powers for hip, knee and ankle (intact limb only) joints when using rigid ( *rig *** **F) and hydraulic ( *hy *** **A-F) ankle-foot devices**

	**Speed Normalised**			**Absolute**		
	***hyA-F***	***rigF***	***p value***	***hyA-F***	***rigF***	***p value***
	Nm/(kg.m/s)			Nm.kg^-1^		
Intact hip moment	+ 0.56 (0.17) / - 0.77 (0.16)	+ 0.61 (0.13) / - 0.82 (0.21)	0.18 / **0.038 (foot)**	+ 0.53 (0.49) / - 0.73 (0.70)	+ 0.56 (0.52) / - 0.76 (0.73)	0.30 / 0.11 (foot)
	+ 0.53 (0.25) / - 0.70 (0.21)	+ 0.64 (0.19) / - 0.88 (0.23)	0.28 /0.30 (speed)	+ 0.61 (0.55) / - 0.82 (0.77)	+ 0.69 (0.63) / - 0.94 (0.90)	0.06 / **0.005 (speed)**
	+ 0.64 (0.22) / - 0.77 (0.16)	+ 0.83 (0.73) / - 1.00 (0.45)	0.71 / 0.35 (interact)	+ 0.88 (0.76) / - 1.05 (0.99)	+ 1.20 (0.73) / - 1.38 (1.10)	0.59 / 0.33 (interact)
Intact knee moment	+ 0.55 (0.22) / - 0.23 (0.20)	+ 0.60 (0.31) / - 0.21 (0.18)	0.17 / 0.29 (foot)	+ 0.54 (0.24) / - 0.20 (0.15)	+ 0.56 (0.31) / - 0.18 (0.15)	0.27 / 0.13 (foot)
	+ 0.47 (0.23) / - 0.16 (0.17)	+ 0.76 (0.14) / - 0.17 (0.17)	0.21 / **< 0.001 (speed)**	+ 0.56 (0.28) / - 0.17 (0.14)	+ 0.79 (0.35) / - 0.18 (0.15)	**0.002** / 0.12 **(speed)**
	+ 0.69 (0.25) / - 0.15 (0.17)	+ 0.69 (0.26) / - 0.13 (0.19)	0.09 / 0.28 (interact)	+ 0.96 (0.43) / - 0.18 (0.16)	+ 0.96 (0.46) / - 0.15 (0.17)	0.11 / 0.40 (interact)
Intact ankle moment	+ 1.29 (0.38) / - 0.14 (0.05)	+ 1.32 (0.39) / - 0.18 (0.04)	0.11 / **0.044 (foot)**	+ 1.20 (0.30) / - 0.13 (0.05)	+ 1.19 (0.22) / - 0.16 (0.04)	0.54 / 0.28 (foot)
	+ 1.13 (0.37) / - 0.19 (0.06)	+ 1.27 (0.47) / - 0.18 (0.09)	**< 0.001** / 0.28 **(speed)**	+ 1.30 (0.28) / - 0.22 (0.07)	+ 1.31 (0.31) / - 0.20 (0.09)	0.35 / **0.004 (speed)**
	+ 0.95 (0.30) / - 0.15 (0.03)	+ 0.92 (0.37) / - 0.16 (0.04)	0.10 / 0.07 (interact)	+ 1.27 (0.35) / - 0.20 (0.04)	+ 1.21 (0.37) / - 0.22 (0.41)	0.52 / 0.06 (interact)
Residual hip moment	+ 0.37 (0.32) / - 0.62 (0.37)	+ 0.41 (0.25) / - 0.64 (0.32)	0.21 / 0.14 (foot)	+ 0.33 (0.25) / - 0.57 (0.32)	+ 0.37 (0.20) / - 0.59 (0.29)	0.26 / 0.43 (foot)
	+ 0.36 (0.32) / - 0.60 (0.36)	+ 0.43 (0.27) / - 0.66 (0.34)	0.76 / 0.65 (speed)	+ 0.40 (0.31) / - 0.68 (0.35)	+ 0.44 (0.24) / - 0.69 (0.30)	**0.016** / **< 0.001 (speed)**
	+ 0.37 (0.28) / - 0.58 (0.35)	+ 0.39 (0.27) / - 0.63 (0.32)	0.48 / 0.27 (interact)	+ 0.47 (0.31) / - 0.75 (0.39)	+ 0.48 (0.27) / - 0.81 (0.30)	0.59 / 0.44 (interact)
Residual knee moment	+ 0.04 (0.02) / - 0.02 (0.01)	+ 0.04 (0.02) / - 0.01 (0.01)	0.47 / 0.09 (foot)	+ 0.03 (0.01) / - 0.02 (0.01)	+ 0.03 (0.01) / - 0.01 (0.01)	0.76 / 0.78 (foot)
	+ 0.04 (0.02) / - 0.01 (0.02)	+ 0.04 (0.02) / - 0.01 (0.01)	**0.021** / **0.049 (speed)**	+ 0.04 (0.01) / - 0.01 (0.01)	+ 0.05 (0.02) / - 0.01 (0.01)	**< 0.001** / 0.84 **(speed)**
	+ 0.03 (0.02) / - 0.02 (0.01)	+ 0.03 (0.01) / - 0.01 (0.00)	0.20 / 0.18 (interact)	+ 0.06 (0.02) / - 0.02 (0.01)	+ 0.05 (0.02) / - 0.02 (0.01)	0.51 / 0.35 (interact)
	W/(kg.m/s)			W.kg^-1^		
Intact hip power	+ 1.51 (0.90) / - 0.43 (0.32)	+ 1.68 (0.98) / - 0.40 (0.14)	0.13 / 0.24 (foot)	+ 0.73 (0.22) / - 0.39 (0.23)	+ 0.78 (0.18) / - 0.37 (0.14)	0.25 / 0.24 (foot)
	+ 1.68 (0.67) / - 0.36 (0.17)	+ 1.99 (1.01) / - 0.47 (0.25)	0.53 / 0.11 (speed)	+ 0.91 (0.26) / - 0.42 (0.21)	+ 1.04 (0.34) / - 0.51 (0.29)	0.12 / **0.036 (speed)**
	+ 1.28 (0.76) / - 0.50 (0.16)	+ 1.26 (0.75) / - 0.77 (0.65)	0.53 / 0.24 (interact)	+ 1.15 (0.51) / - 0.67 (0.24)	+ 1.66 (1.83) / - 1.12 (1.20)	0.46 / 0.27 (interact)
Intact knee power	+ 0.38 (0.17) / - 0.92 (0.24)	+ 0.38 (0.23) / - 0.98 (0.39)	0.09 / 0.08 (foot)	+ 0.36 (0.18) / - 0.91 (0.35)	+ 0.35 (0.23) / - 0.93 (0.43)	0.13 / 0.13 (foot)
	+ 0.37 (0.16) / - 0.84 (0.38)	+ 0.60 (0.37) / - 1.31 (0.56)	**< 0.001** / 0.25 **(speed)**	+ 0.44 (0.21) / - 1.02 (0.51)	+ 0.63 (0.35) / - 1.41 (0.58)	**< 0.001** / **< 0.001 (speed)**
	+ 0.62 (0.31) / - 1.03 (0.32)	+ 0.69 (0.25) / - 1.12 (0.37)	0.11 / 0.06 (interact)	+ 0.87 (0.55) / - 1.46 (0.61)	+ 0.96 (0.46) / - 1.55 (0.63)	0.16 / 0.09 (interact)
Intact ankle power	+ 1.51 (0.90) / - 0.74 (0.27)	+ 1.68 (0.98) / - 0.79 (0.29)	0.30 / 0.10 (foot)	+ 1.47 (0.90) / - 0.70 (0.24)	+ 1.55 (0.91) / - 0.72 (0.24)	0.74 / 0.44 (foot)
	+ 1.68 (0.67) / - 0.77 (0.48)	+ 1.99 (1.01) / - 0.87 (0.53)	**0.048** / 0.21 **(speed)**	+ 1.93 (0.80) / - 0.92 (0.68)	+ 2.09 (0.91) / - 0.95 (0.70)	0.14 / 0.49 (speed)
	+ 1.28 (0.75) / - 0.54 (0.27)	+ 1.26 (0.75) / - 0.56 (0.29)	0.47 / 0.41 (interact)	+ 1.85 (1.27) / - 0.72 (0.35)	+ 1.80 (1.29) / - 0.74 (0.38)	0.82 / 0.95 (interact)
Residual hip power	+ 0.67 (0.38) / - 0.49 (0.35)	+ 0.72 (0.37) / - 0.67 (0.25)	0.63 / 0.58 (foot)	+ 0.63 (0.26) / - 0.45 (0.28)	+ 0.65 (0.30) / - 0.53 (0.24)	0.93 / 0.82 (foot)
	+ 0.72 (0.44) / - 0.57 (0.47)	+ 0.73 (0.36) / - 0.58 (0.35)	0.89 / 0.66 (speed)	+ 0.81 (0.39) / - 0.64 (0.48)	+ 0.76 (0.28) / - 0.62 (0.34)	**< 0.001** / 0.08 **(speed)**
	+ 0.69 (0.40) / - 0.54 (0.41)	+ 0.74 (0.41) / - 0.53 (0.38)	0.74 / 0.41 (interact)	+ 0.89 (0.39) / - 0.69 (0.49)	+ 0.94 (0.41) / - 0.67 (0.43)	0.34 / 0.30 (interact)
Residual knee power	+ 0.10 (0.15) / - 0.46 (0.50)	+ 0.06 (0.07) / - 0.41 (0.44)	0.11 / 0.07 (foot)	+ 0.10 (0.15) / - 0.43 (0.48)	+ 0.05 (0.05) / - 0.37 (0.40)	0.10 / 0.08 (foot)
	+ 0.10 (0.12) / - 1.05 (1.12)	+ 0.04 (0.03) / - 0.42 (0.45)	0.76 / **0.017 (speed)**	+ 0.11 (0.14) / - 1.20 (1.34)	+ 0.04 (0.02) / - 0.42 (0.41)	0.99 / **0.025 (speed)**
	+ 0.09 (0.15) / - 0.56 (0.69)	+ 0.03 (0.02) / - 0.42 (0.46)	0.90 / **0.034 (interact)**	+ 0.12 (0.20) / - 0.79 (1.02)	+ 0.04 (0.02) / - 0.58 (0.67)	0.83 / **0.036 (interact)**

Values normalised to walking speed are in the left-hand columns and absolute values in the right-hand columns. Results are listed downwards - slow, customary speed and fast. Statistically significant differences are in **bold**.

**Table 2:** **Group mean (± SD) positive, negative and total stance-phase work done at the hip, knee and ankle (intact limb only) joints when using rigid ( *rig *** **F) and hydraulic ( *hy *** **A-F) ankle-foot devices**

	**Speed normalised**			**Absolute**		
	***hyA-F***	***rigF***	***p value***	***hyA-F***	***rigF***	***p value***
	J/(kg.m/s)			J.kg^-1^		
Intact hip total work	0.21 (0.12)	0.25 (0.09)	0.19 (foot)	0.24 (0.11)	0.24 (0.07)	0.36 (foot)
	0.24 (0.11)	0.26 (0.11)	**0.014 (speed)**	0.25 (0.11)	0.27 (0.12)	**0.027 (speed)**
	0.29 (0.15)	0.36 (0.18)	0.57 (interact)	0.36 (0.13)	0.45 (0.36)	0.44 (interact)
Intact hip positive work	+0.18 (0.11)	+0.18 (0.08)	0.74 (foot)	+0.17 (0.08)	+0.16 (0.07)	0.73 (foot)
	+0.16 (0.10)	+0.16 (0.09)	0.42 (speed)	+0.18 (0.09)	+0.17 (0.08)	0.09 (speed)
	+0.17 (0.11)	+0.20 (0.15)	0.41 (interact)	+0.22 (0.11)	+0.27 (0.24)	0.41 (interact)
Intact hip negative work	-0.08 (0.04)	-0.08 (0.04)	0.21 (foot)	-0.07 (0.04)	-0.08 (0.05)	0.20 (foot)
	-0.06 (0.03)	-0.10 (0.07)	0.12 (speed)	-0.07 (0.05)	-0.10 (0.08)	**0.01 (speed)**
	-0.10 (0.05)	-0.13 (0.08)	0.41 (interact)	-0.14 (0.06)	-0.18 (0.14)	0.48 (interact)
Intact knee total work	0.21 (0.06)	0.22 (0.11)	0.18 (foot)	0.21 (0.08)	0.17 (0.09)	0.46 (foot)
	0.18 (0.08)	0.27 (0.12)	**0.03 (speed)**	0.21 (0.11)	0.20 (0.12)	**< 0.001 (speed)**
	0.26 (0.10)	0.28 (0.10)	0.09 (interact)	0.36 (0.16)	0.38 (0.11)	0.09 (interact)
Intact knee positive work	+0.06 (0.03)	+0.06 (0.03)	0.19 (foot)	+0.06 (0.03)	+0.06 (0.03)	0.30 (foot)
	+0.06 (0.03)	+0.09 (0.05)	**0.030 (speed)**	+0.06 (0.04)	+0.09 (0.05)	**0.001 (speed)**
	+0.08 (0.04)	+0.09 (0.04)	0.06 (interact)	+0.12 (0.06)	+0.12 (0.06)	0.07 (interact)
Intact knee negative work	-0.15 (0.04)	-0.16 (0.09)	0.21 (foot)	-0.14 (0.06)	-0.15 (0.09)	0.27 (foot)
	-0.12 (0.06)	-0.18 (0.08)	0.07 (speed)	-0.15 (0.08)	-0.20 (0.08)	**< 0.001 (speed)**
	-0.18 (0.06)	-0.19 (0.06)	0.21 (interact)	-0.25 (0.10)	-0.27 (0.11)	0.25 (interact)
Intact ankle total work	0.34 (0.18)	0.37 (0.20)	**0.032 (foot)**	0.32 (0.12)	0.33 (0.17)	0.25 (foot)
	0.35 (0.18)	0.39 (0.21)	**0.011 (speed)**	0.40 (0.19)	0.40 (0.19)	0.08 (speed)
	0.24 (0.11)	0.26 (0.13)	0.42 (interact)	0.33 (0.17)	0.36 (0.22)	0.64 (interact)
Intact ankle positive work	+0.17 (0.12)	+0.19 (0.14)	0.17 (foot)	+0.17 (0.12)	+0.17 (0.12)	0.54 (foot)
	+0.20 (0.11)	+0.22 (0.13)	**0.036 (speed)**	+0.23 (0.12)	+0.23 (0.13)	0.10 (speed)
	+0.13 (0.08)	+0.14 (0.11)	0.89 (interact)	+0.19 (0.13)	+0.21 (0.19)	0.67 (interact)
Intact ankle negative work	-0.17 (0.07)	-0.18 (0.08)	**0.003 (foot)**	-0.15 (0.06)	-0.16 (0.06)	0.15 (foot)
	-0.15 (0.09)	-0.17 (0.09)	**0.024 (speed)**	-0.17 (0.09)	-0.17 (0.08)	0.63 (speed)
	-0.11 (0.05)	-0.12 (0.05)	0.24 (interact)	-0.15 (0.06)	-0.13 (0.07)	0.98 (interact)
Residual hip total work	0.22 (0.19)	0.21 (0.17)	0.44 (foot)	0.20 (0.14)	0.19 (0.14)	**0.004 (foot)**
	0.22 (0.19)	0.22 (0.18)	0.68 (speed)	0.24 (0.17)	0.22 (0.16)	**0.003 (speed)**
	0.24 (0.17)	0.23 (0.16)	0.82 (interact)	0.31 (0.16)	0.27 (0.16)	0.28 (interact)
Residual hip positive work	+0.15 (0.17)	+0.15 (0.14)	0.75 (foot)	+0.14 (0.12)	+0.13 (0.12)	0.57 (foot)
	+0.15 (0.16)	+0.15 (0.14)	0.92 (speed)	+0.17 (0.14)	+0.15 (0.12)	**0.009 (speed)**
	+0.15 (0.14)	+0.16 (0.13)	0.51 (interact)	+0.19 (0.14)	+0.19 (0.12)	**0.046 (interact)**
Residual hip negative work	-0.07 (0.04)	-0.06 (0.04)	0.26 (foot)	-0.06 (0.04)	-0.06 (0.04)	0.14 (foot)
	-0.07 (0.05)	-0.07 (0.06)	0.26 (speed)	-0.08 (0.06)	-0.07 (0.07)	**0.002 (speed)**
	-0.08 (0.05)	-0.07 (0.05)	0.43 (interact)	-0.11 (0.07)	-0.09 (0.07)	0.45(interact)
Residual knee total work	0.08 (0.09)	0.08 (0.09)	0.052 (foot)	0.13 (0.14)	0.09 (0.09)	**0.044 (foot)**
	0.10 (0.09)	0.07 (0.08)	0.62 (speed)	0.13 (0.15)	0.09 (0.10)	**< 0.001 (speed)**
	0.08 (0.09)	0.06 (0.06)	0.32 (interact)	0.21 (0.24)	0.13 (0.14)	0.32 (interact)
Residual knee positive work	+0.01 (0.01)	+0.006 (0.01)	0.91 (foot)	+0.005 (0.01)	+0.005 (0.01)	0.99 (foot)
	+0.007 (0.01)	+0.003 (0.00)	0.90 (speed)	+0.006 (0.01)	+0.003 (0.00)	0.47 (speed)
	+0.005 (0.01)	+0.007 (0.01)	0.35 (interact)	+0.007 (0.01)	+0.010 (0.01)	0.36(interact)
Residual knee negative work	-0.08 (0.09)	-0.07 (0.09)	**0.036 (foot)**	-0.07 (0.09)	-0.07 (0.08)	**0.025 (foot)**
	-0.09 (0.09)	-0.06 (0.07)	0.48 (speed)	-0.10 (0.09)	-0.06 (0.07)	0.45 (speed)
	-0.07 (0.09)	-0.05 (0.06)	0.29 (interact)	-0.10 (0.13)	-0.07 (0.08)	0.19 (interact)
Intact limb (all joints) total work	0.76 (0.21)	0.84 (0.28)	**0.047 (foot)**	0.73 (0.24)	0.77 (0.27)	0.15 (foot)
	0.77 (0.27)	0.92 (0.24)	0.36 (speed)	0.88 (0.28)	0.97 (0.33)	**< 0.001 (speed)**
	0.80 (0.25)	0.90 (0.26)	0.50 (interact)	1.08 (0.38)	1.24 (0.67)	0.51 (interact)
Residual limb (all joints) total work	0.30 (0.20)	0.29 (0.17)	0.12 (foot)	0.27 (0.16)	0.26 (0.15)	0.024 (foot)
	0.32 (0.24)	0.28 (0.18)	0.96 (speed)	0.35 (0.22)	0.29 (0.16)	**< 0.001 (speed)**
	0.32 (0.18)	0.29 (0.17)	0.48 (interact)	0.41 (0.19)	0.36 (0.18)	0.11 (interact)

Results are listed downwards - slow, customary speed and fast. Values normalised to walking speed are in the left-hand columns and absolute values in the right-hand columns. Statistically significant differences are in **bold**.

**Table 3:** **Group mean (± SD) stance phase moments and power integrals at distal end of prosthetic shank ( *pros-end *) when using rigid ( *rig *** **F) and hydraulic ( *hy *** **A-F) ankle-foot devices**

	**Speed Normalised**			**Absolute**		
	***hyA-F***	***rigF***	***p value***	***hyA-F***	***rigF***	***p value***
	Nm.s/(kg.m/s)			Nm.s.kg^-1^		
External early-stance 'plantarflexion’ moment impulse	-0.06 (0.03)	-0.06 (0.03)	0.36 (foot)	-0.05 (0.03)	-0.06 (0.03)	0.54 (foot)
	-0.05 (0.02)	-0.06 (0.02)	0.13 (speed)	-0.07 (0.03)	-0.07 (0.02)	**0.028 (speed)**
	-0.05 (0.02)	-0.05 (0.02)	0.81 (interact)	-0.06 (0.03)	-0.07 (0.02)	0.058 (interact)
External mid/late stance 'dorsiflexion’ moment impulse	+0.43 (0.14)	+0.46 (0.13)	0.30 (foot)	+0.40 (0.13)	+0.42 (0.07)	0.79 (foot)
	+0.31 (0.12)	+0.34 (0.10)	**<0.001 (speed)**	+0.36 (0.09)	+0.36 (0.06)	**<0.001 (speed)**
	+0.27 (0.11)	+0.27 (0.08)	0.12 (interact)	+0.35 (0.09)	+0.34 (0.05)	0.12 (interact)
	J/(kg.m/s)			J.kg^-1^		
Negative work – early stance	-0.02 (0.01)	-0.01 (0.01)	**0.009 (foot)**	-0.02 (0.01)	-0.02 (0.01)	**0.011 (foot)**
	-0.03 (0.01)	-0.02 (0.01)	**0.017 (speed)**	-0.03 (0.01)	-0.02 (0.01)	**0.005 (speed)**
	-0.02 (0.01)	-0.02 (0.01)	0.55 (interact)	-0.03 (0.01)	-0.03 (0.01)	0.21 (interact)
Positive work - early stance	+0.003 (0.00)	+0.003 (0.00)	**< 0.001 (foot)**	+0.004 (0.00)	+0.004 (0.00)	**0.011 (foot)**
	+0.003 (0.00)	+0.006 (0.00)	**0.007 (speed)**	+0.003 (0.00)	+0.007 (0.00)	**< 0.001 (speed)**
	+0.008 (0.00)	+0.010 (0.00)	0.09 (interact)	+0.012 (0.00)	+0.014 (0.01)	0.09 (interact)
Negative work - mid-stance	-0.11 (0.05)	-0.10 (0.05)	0.08 (foot)	-0.15 (0.04)	-0.12 (0.04)	**0.043 (foot)**
	-0.14 (0.07)	-0.13 (0.04)	0.36 (speed)	-0.16 (0.05)	-0.13 (0.03)	0.39 (speed)
	-0.11 (0.04)	-0.11 (0.04)	0.48 (interact)	-0.14 (0.04)	-0.14 (0.03)	0.13 (interact)
Positive work - late stance	+0.07 (0.03)	+0.08 (0.03)	**< 0.001 (foot)**	+0.09 (0.03)	+0.11 (0.03)	**< 0.001 (foot)**
	+0.08 (0.03)	+0.11 (0.03)	0.47 (speed)	+0.09 (0.03)	+0.12 (0.03)	0.47 (speed)
	+0.06 (0.03)	+0.09 (0.02)	**0.024 (interact**)	+0.09 (0.03)	+0.12 (0.04)	**0.027 (interact**)
Total work	0.20 (0.08)	0.19 (0.07)	0.22 (foot)	0.19 (0.06)	0.17 (0.05)	0.84 (foot)
	0.25 (0.09)	0.26 (0.08)	0.20 (speed	0.29 (0.07)	0.28 (0.06)	**0.023 (speed)**
	0.20 (0.06)	0.22 (0.06)	0.09 (interact)	0.29 (0.12)	0.31 (0.11)	0.10 (interact)
Energy dissipated (negative + positive work)	0.06 (0.04)	0.03 (0.03)	**0.001 (foot)**	0.08 (0.03)	0.03 (0.04)	**< 0.001 (foot)**
	0.08 (0.06)	0.03 (0.02)	0.71 (speed)	0.10 (0.05)	0.03 (0.03)	**0.046** (speed)
	0.07 (0.04)	0.03 (0.02)	0.43 (interact)	0.08 (0.04)	0.03 (0.03)	0.25 (interact)
Time of moment ’flip’ (% stance)	28.5 (4.9)	32.9 (3.8)	**0.029 (foot)**			
	30.8 (5.3)	36.1 (5.9)	0.25 (speed)			
	28.4 (4.8)	34.0 (4.1)	0.31 (interact)			

Results are listed downwards - slow, customary speed and fast. Values normalised to walking speed are in the left-hand columns and absolute values in the right-hand columns. Statistically significant differences are in **bold**.

### Intact limb hip, knee and ankle

The intact limb hip peak flexion moment was significantly affected by attachment category (*p* = 0.038), and was reduced (all speeds) when using the *hy*A-F. There was no significant effect of attachment category or interaction between attachment category and speed level on the joint kinetics at the intact knee. The intact limb peak dorsiflexion moment was significantly affected by attachment category (*p* = 0.044), and was lower when using the *hy*A-F (all speeds). The intact ankle negative work (*p* = 0.032) and total work (*p* = 0.003) done was significantly affected by attachment category; less work was done when using the *hy*A-F. There was also a significant effect of attachment category on the total joint work done by the intact limb (summation of work done across all joints, *p* = 0.047); indicating reduced work when using the *hy*A-F.

### Residual limb hip and knee

There was no significant effect of attachment category or interaction between attachment category and speed level on the joint kinetics at the residual hip. The negative power peak at the residual knee was affected by a speed level-by-attachment category interaction (*p* ≤ 0.034): peak power was higher using the *hy*A-F at customary speed compared to all other conditions (*p* ≤ 0.016). Residual knee negative work was affected by attachment category (*p* = 0.047); indicating increased work when using the *hy*A-F.

### Prosthetic 'ankle’

The timing of when the external moment at the *pros end* changed direction (sign) was affected by attachment category (*p* = 0.029). The moment changed from one tending to plantarflex to one tending to dorsiflex earlier, at 29% compared to 34% stance phase, when using the *hy*A-F. The negative power integral in early stance was significantly affected by attachment category (*p* = 0.009); indicating more energy flowed from the shank to the foot (energy absorption) at all speed levels when using the *hy*A-F. The positive power integral during early stance (energy return) was also significantly affected by attachment category (*p* < 0.001); indicating less energy flowed into the shank from the foot when using the *hy*A-F. Despite there being no difference between attachment conditions in the negative power integral during mid-stance (*p* = 0.08), the positive power integral during late stance was affected by attachment category (*p* < 0.001) and by a speed level-by-attachment category interaction (*p* = 0.024). There was less energy flow into the shank from the foot when using the *hy*A-F, at all speeds (*p* ≤ 0.019), with greater reduction at customary speed compared to both slow and fast speeds.

## Discussion

The objective of this study was to compare speed-related joint kinetic adaptations when using the *hy*A-F compared to *rig*F. The speed of walking at all three freely chosen speed levels was higher when using the *hy*A-F (although only significantly so for customary speed walking), and thus to make the comparison of attachment conditions more balanced / equitable, joint kinetic data were evaluated both normalised to walking speed and in absolute terms.

While there were no differences between attachment categories in the amount of compensatory joint work at the intact limb hip (absolute or normalised), the normalised peak power generation and total joint work at the intact limb ankle were reduced at all speed levels when using the *hy*A-F. Therefore our hypothesis that the compensatory joint power generation at the intact limb hip and ankle would be reduced through use of the *hy*A-F was only partially supported.

It is noteworthy that in absolute terms there was no difference between attachment categories in total work at the intact ankle across all speed levels (*hy*A-F 0.353 ± 0.173 Jkg^-1^, *rig*F 0.365 ± 0.189 Jkg^-1^, *p* = 0.25) but as walking speeds were higher when using the *hy*A-F, this resulted in there being a reduction in total ankle work done per meter travelled. The reduction in peak power generation during late stance at the intact ankle when using the *hy*A-F could possibly be a result of there being less resistance to forwards progression in early stance on the prosthetic limb because the prosthetic shank was able to rotate forwards more easily during this period [19]. Thus, for the same amount of mechanical effort at the intact limb ankle (and hip) there was greater COM progression.

At the residual knee the increased power absorption peak at customary speed when using the *hy*A-F and significant increase in (normalised and absolute) negative, eccentric work at all speed levels, indicate the residual knee was more active / involved during weight bearing when using the *hy*A-F, particularly so at the customary speed. To gain further insight into the increased residual knee loading / involvement when using the *hy*A-F we retrospectively examined knee flexion during loading response, which is typically reported to be reduced on the residual side in trans-tibial amputees [14,37], along with the GRF and residual knee joint reaction force (normalised to body weight, BW) during prosthetic-limb stance. There was significantly more angular displacement during loading response at the intact knee compared to the residual knee (intact ~22°, residual ~6°, *p* = 0.001), but there was no difference in residual knee angular displacement during loading response across attachment categories or speed levels (*p* ≥ 0.49). However, the residual knee was in a more flexed position at initial contact (and thus throughout loading response) when using the *hy*A-F compared to *rig*F, and differences in knee flexion angle (at initial contact) between attachment categories increased with speed level (slow *hy*A-F 8.2° ± 3.2°, *rig*F 5.1° ± 6.2°; customary *hy*A-F 6.5° ± 3.9°, *rig*F 3.7° ± 6.9°; fast *hy*A-F 10.0° ± 5.0°, *rig*F 4.3° ± 6.5°, *p* = 0.019). This change in limb posture at initial contact could have been due to an increased 'dorsiflexion’ angle at toe-off (due to the articulation provided by the hydraulic device), which brought the shank forwards, or due to a drive to allow the knee to be loaded more because of perceived increased comfort (previous research has shown that in-socket pressures are reduced when using the *hy*A-F [18]; which suggests increased comfort levels). When the *hy*A-F was used there was a significant increase in both the peak vertical GRF (*hy*A-F, 1.05 ± 0.14 BW, *rig*F, 1.02 ± 0.14 BW, *p* = 0.007) and the vertical GRF impulse (*hy*A-F, 0.60 ± 0.09 BW, *rig*F, 0.57 ± 0.09 BW.s, *p* = 0.001), accompanied by a significant increase in the axial (relative to the shank) residual knee peak joint reaction force (*hy*A-F, 1.15 ± 0.18 BW, *rig*F, 1.11 ± 0.017 BW, *p* = 0.044), indicative of increased weight bearing. Consequently, our hypothesis that the residual knee would be loaded more during weight bearing was supported. Previous research has reported the power absorption peak to be significantly reduced at the residual knee compared to that at the intact knee [11,14]. Thus the findings of the present study suggest that the hydraulic device had an important and clinically meaningful effect on how the residual knee was loaded. It is worth emphasising however, that no statistical comparisons were made between the joint kinetics of the intact and residual knees due to the differing modelling approaches used for each limb. In addition, the magnitude of the moments and powers at the residual knee were very small in comparison to values at the intact knee.

At the *pros end*, net power flow presented a double bi-phasic pattern of power absorption and return irrespective of attachment category and across all speed levels (Figure 4). Such a double bi-phasic pattern was reported by Prince et *al*. [28]. These two periods must have corresponded respectively with the heel and then the forefoot keel elastically deforming and recoiling. While compression of the heel keel following initial contact plays a role in increasing comfort and allowing the prosthetic forefoot to lower to the ground [38] the energy return, during early-to-mid stance, associated with its subsequent recoil is an unusual phenomenon when compared to intact ankle joint kinetics. Such energy return could not have directly contributed to gait propulsion as it occurred at approximately 20% of stance, and indeed may have affected gait inappropriately / negatively; potentially causing an early heel rise and / or a 'bouncing’ or unstable sensation. This inappropriate energy return from the foot, which increased with speed level for both attachment categories, was significantly reduced at all speed levels when participants used a *hy*A-F (normalised and absolute). The change from inappropriate energy return (recoil) to absorption was synchronous with the external moment switching from one tending to plantar-flex to one tending to dorsi-flex. This moment 'flip’ occurred approximately 5% earlier in stance at all speed levels when using the *hy*A-F (i.e. at 29% of stance compared to 34% of stance when using the *rig*F), and occurred at a similar time to that reported previously for overground gait in trans-tibial amputees (~30% of stance [39,40]) but later than that seen in able-bodied gait (~9% of stance [3]). The earlier moment 'flip’ when using the *hy*A-F may have been due to the device preventing the COP trajectory 'stalling’ under the prosthetic hindfoot during its progression from the heel to the toe region [19]. In turn this resulted in the CoP passing anterior to the *pros end* sooner. With the increase in power absorption and reduction in power return more energy was dissipated by the *hy*A-F across all speed levels. Such energy dissipation is a feature of hydraulic damping. Likely as a result of such damping, many of the participants commented that when using the *hy*A-F they no longer felt they had to “climb over” the prosthetic foot or commented they felt less resistance to forward progression. A feeling of improved ability to 'get onto and over’ the prosthetic foot suggests that the *hy*A-F attenuates / reduces the 'braking’ effect the foot conveys to COM progression. This would further explain why walking speed increased and there was a reduction in intact limb joint work done per meter travelled when using the device. A reduction in work per meter travelled suggests use of a *hy*A-F may potentially result in a reduction in metabolic energy costs, and this should be investigated in future work.

Regardless of speed level or attachment category, during early stance the magnitude of P_rot_ (positive) was smaller than that of P_trans_ (negative) yielding a net negative power flow as the heel deformed (Figure 1). Once the foot became plantigrade, P_rot_ increased relative to P_trans_ (Figure 1) as the distal end of the shank became the fulcrum of shank rotation above the foot. At this time there was a net positive power flow which must have been due to the recoil of the heel keel. However, when the *hy*A-F was used the earlier moment 'flip’, and relative increase in P_rot_, tended to reduce this period of positive power flow at all speed levels. When using the *hy*A-F the heel keel would have still recoiled but some of the energy returned was likely dissipated within the hydraulic unit rather than being transferred to the shank segment. As a result there was almost continuous negative power flow for the first three-quarters of stance (Figure 4). The earlier moment 'flip’ when using the hyA-F signified that the passive control exerted on the forward rotating shank (external moment tending to dorsiflex) occurred sooner when using the device which is consistent with the increased time of negative power flow. This indicates the device provided 'ankle’ function that was more akin to that typical of able-bodied gait [1-3].

There were no significant differences in the normalised negative power associated with forefoot compression (i.e. during mid-to-late stance period) across speed levels or between attachment categories. This is, perhaps, unsurprising given that the hydraulic device reaches its limit of articulation during mid-stance and thus the behaviour of the foot in late stance should be similar for both attachment categories. Having said this, the positive power flow as the forefoot keel recoiled, which mimics the A2 power burst seen in able-bodied gait, was significantly reduced at all walking speed levels when using the *hy*A-F (normalised and absolute). There is no obvious explanation for this, but it is possible that the increased negative work at the residual knee facilitated power flow into the shank from the thigh, which in turn may have contributed to the net power flow observed at the *pros end*.

The rate at which the hydraulic unit articulates during stance is a function of walking speed and level of hydraulic damping. This means the 'setting up’ process conducted by the prosthetist is paramount for optimal functioning of the device. The late stance power flow into the shank was higher at customary walking speed than the other speed levels when using the *hy*A-F, which suggests its time-dependent nature contributed indirectly to the energy returned by the prosthetic foot. The differences observed in the energy absorbed, and dissipated or returned, by the *hy*A-F and *rig*F were of a much smaller magnitude than the kinetic changes observed on the intact limb. This highlights how small alterations of a prosthetic device can have profound effects further along the kinetic chain.

Irrespective of attachment category, the normalised peak power generation and total joint work at the intact ankle became reduced as speed level increased, but there were simultaneous increases in (normalised and absolute) total joint work at the intact knee and hip. These speed effects are similar to those from previous studies investigating speed-related joint kinetics changes in able-bodied gait [15,16]. As knee kinetics play a limited role in gait propulsion [4], our findings suggest that amputees, like able-bodied individuals, predominantly alter hip kinetics in order to increase walking speed. In the present study there was no significant increase with speed level in the normalised residual hip peak power absorption, generation or total joint work, indicating that amputees relied predominantly on the intact limb hip for increased propulsion, regardless of attachment condition. These findings are consistent with the findings of Powers et *al*. [14] who reported residual limb hip peak power generation to be lower than that for the intact limb hip at customary speed walking.

The method used in the present study to estimate power flow at the *pros end* only considered sagittal plane kinetics and thus under estimated the total power flow to and from the prosthetic foot. However, as our primary aim was to determine whether use of an articulating hydraulic device lessened speed-related adaptations in joint kinetics, this limitation was deemed acceptable. In essence the approach adopted determined the energy absorbed and returned by the prosthetic foot that contributed to gait propulsion, and was thus in keeping with the approach we used to determine sagittal plane muscle moments and powers at all physiological lower-limb joints. To undertake an evaluation of absolute power absorbed and returned by a dynamic response foot, a different approach is recommended [41]. Similarly, determining total mechanical work done by a physiological joint (rather than just that associated with muscle work; as was carried out in the present study), also requires a different approach [42].

## Conclusion

Findings indicate that a trans-tibial prosthesis incorporating a dynamic-response foot attached via an articulating hydraulic device reduced the speed-related increases in compensatory intact-limb joint kinetics compared to when the foot was attached rigidly. In addition, residual knee loading / involvement and weight bearing on the prosthetic side were increased when using the *hy*A-F. Differences between attachment types were highest at the customary speed level which indicates the hydraulic ankle-foot device, like other passive prosthetic devices, is most effectual at the walking speed it is set up for. Finally, the observed reduction in intact-limb ankle and combined joints work per metre travelled when using the hyA-F, occurred despite the hyA-F dissipating more energy during stance. This suggests that 'energy return’ *per se* is not necessarily the key design criterion for a prosthetic foot.

## Abbreviations

BW: Body weight; COM: Centre of mass; CoP: Centre-of-pressure; GRF: Ground reaction force; hyA-F: dynamic-response foot with hydraulic ankle attachment; pros end: Distal end of the prosthetic shank; rigF: dynamic-response foot with rigid ankle attachment; UTA: Unilateral trans-tibial amputee.

## Competing interests

The authors declare that they have no competing interests.

## Authors’ contributions

Conceived and designed the experiments; ADA, RM, JK, JB. Oversaw participant recruitment; RM. Performed the experiments and analysed the data; ADA, JB. Wrote the manuscript; ADA, JB. All authors read and approved the final manuscript.
